# Prioritizing suicide prevention guideline recommendations in specialist mental healthcare: a Delphi study

**DOI:** 10.1186/s12888-020-2465-0

**Published:** 2020-02-07

**Authors:** Kim Setkowski, Anton J. L. M. van Balkom, Dave A. Dongelmans, Renske Gilissen

**Affiliations:** 1 113 Suicide Prevention, Amsterdam, The Netherlands; 2 Department of Psychiatry, Amsterdam UMC, location VUmc, Amsterdam Public Health research institute and GGZ inGeest, Amsterdam, The Netherlands; 3 Department of Intensive Care Medicine, Amsterdam UMC, location AMC, Amsterdam, The Netherlands; 4 National Intensive Care Evaluation (NICE) foundation, Amsterdam, The Netherlands

**Keywords:** Suicide prevention, Implementation, Guideline recommendations, Mental healthcare, Delphi study, Quality indicator

## Abstract

**Background:**

The Delphi technique is a proven and reliable method to create common definitions and to achieve convergence of opinion. This study aimed to prioritize suicide prevention guideline recommendations and to develop a set of quality indicators (QIs) for suicide prevention in specialist mental healthcare.

**Methods:**

This study selected 12 key recommendations from the guideline to modify them into QIs. After feedback from two face-to-face workgroup sessions, 11 recommendations were rephrased and selected to serve as QIs. Next, a Delphi study with the 11 QIs was performed to achieve convergence of opinion among a panel of 90 participants (23 suicide experts, 23 members of patients’ advisory boards or experts with experiences in suicidal behavior and 44 mental healthcare professionals). The participants scored the 11 QIs on two selection criteria: relevance (it affects the number of suicides in the institution) and action orientation (institutions or professionals themselves can influence it) using a 5-point Likert scale. Also, data analysts working in mental healthcare institutions (MHIs) rated each QI on feasibility (is it feasible to monitor and extract from existing systems). Consensus was defined as 70% agreement with priority scores of four or five.

**Results:**

Out of the 11 recommendations, participants prioritized five recommendations as relevant and action-oriented in optimizing the quality of care for suicide prevention: 1) screening for suicidal thoughts and behavior, 2) safety plan, 3) early follow-up on discharge, 4) continuity of care and 5) involving family or significant others. Only one of the 11 recommendations early follow-up on discharge reached consensus on all three selection criteria (relevance, action orientation, and feasibility).

**Conclusions:**

The prioritization of relevant and action-oriented suicide prevention guideline recommendations is an important step towards the improvement of quality of care in specialist mental healthcare.

## Background

In the Netherlands, about 40% of all people who die by suicide are in specialist mental health care [1]. The implementation of suicide prevention guideline recommendations into routine care appears to be an effective strategy to prevent suicides in MHIs. A large-scale study in the United Kingdom showed a significant reduction in suicide rates after implementation of 16 service improvements (including training of clinical staff, policy on the follow-up of discharged patients and ward safety) in MHIs [2]. In the Netherlands, the multidisciplinary guideline for the diagnosis and treatment of suicidal behavior was published in 2012 [3]. This guideline was developed to optimize the care for patients with suicidality in mental healthcare. However, its uptake by the field is varying, resulting in different suicide prevention policies and practices within and between MHIs in the Netherlands [4, 5].

To optimize guideline implementation and reduce the suicide rate in Dutch MHIs, 113 Suicide Prevention (the Dutch expertise center on suicide prevention and lifeline) formed a Suicide Prevention Action Network (SUPRANET) in mental healthcare (www.supranetggz.nl). SUPRANET is a confidential learning network of at present 16 specialist MHIs in the Netherlands. SUPRANET aims at optimizing the quality and safety of care to enhance suicide prevention. This network collects data on suicide and suicide attempts, provides biannual benchmark feedback reports to participant organizations and organizes meetings for the exchange of best-practices [6].

To improve the quality of care and prevent suicides as well as eliminate discrepancies in specialist mental healthcare, it is of great importance to prioritize the guideline recommendations and to define measurable elements designed to evaluate aspects of the quality of care [7–10]. These QIs should be relevant, actionable, reliable, show room for improvement and data collection of the indicator should be feasible [11]. Using QIs to monitor changes in adherence to the suicide prevention guideline in specialist mental healthcare is an essential step towards the delivery of evidence-based care [12].

Because all patients with an increased risk for suicidality should receive evidence-based care within MHIs, the literature should try to underpin the effectiveness of every QI and its relation to reduced suicide (attempt) rates. Up until now, the literature examined the effectiveness of several guideline recommendations [3]. A variety of pharmacological and psychotherapeutic interventions have repeatedly been found effective in the treatment of suicidality [13–17]. For example, a study of Linehan et al. [18] found evidence for the effectiveness of dialectical behavior therapy (DBT) in decreasing the number of suicide attempts as well as reducing the number of hospitalization visits for suicide ideation in women with a borderline personality disorder [19]. To our knowledge, however, not all recommendations from the suicide prevention guideline have been extensively researched in the literature [3]. For example, safety planning is often informed by research and clinical practice as an important guideline recommendation but does not yet have a body of research to support it. Fortunately, the evidence is building. A randomized controlled trial (RCT) by Bryan et al. [20] found a positive association between crisis response planning (an intervention related to safety planning) and reduced suicide attempt rates compared to the use of contracts for safety (e.g., which states that the patient with suicidality should not engage in suicidal behavior during a crisis) in US soldiers. Although several studies have been published on the effectiveness and use of safety planning [21], more research is still needed. A recent publication by Brent, Oquendo, and Reynolds [22] emphasized that formulating a safety plan is one of the seven key elements to treat patients with suicidality effectively.

As suggested by the review of Zalsman et al. [13], no single suicide prevention strategy is way more effective compared to the others. Multiple relevant and action-oriented key recommendations should therefore be implemented at the same time. Also, While et al. [23] showed that mental healthcare services who implement multiple strategies (seven to nine recommendations) at the same time show the most significant reduction in the number of suicides compared to those implementing fewer recommendations.

This study aimed to prioritize the suicide prevention guideline recommendations and to develop a set of relevant and actionable QIs for suicide prevention in specialist mental healthcare. The Delphi technique was used to create common definitions and terminology, and to achieve convergence of opinion among the participants. Participants of this study were suicide experts, health care professionals, and experts with experiences in suicidal behavior and members of patients’ advisory boards. Criteria for selection were 1) relevance (it affects the number of suicides in the institution) and 2) action orientation (the institutions or professionals themselves can influence it). In the last step, data analysts scored the QIs on 3) feasibility to monitor and extract them from existing systems. The Delphi technique is a reliable method in selecting QIs and uses a structured, iterative process of collecting knowledge from a group of experts with the primary goal of reaching consensus [24–26].

## Methods

As part of the SUPRANET study [6], a Delphi study was done to prioritize the guideline recommendations and to select and standardize the terminology of QIs. This study has been approved by the Central Committee on Research Involving Human Subjects in the Netherlands (CCMO) and does not fall under the scope of the Medical Research Involving Human Subjects Act (WMO). The CCMO states that: “In general, research with human subjects only falls under the Medical Research (Human Subjects) Act (WMO) if there is an infringement of the physical and/or psychological integrity of the subject” (https://english.ccmo.nl/investigators/legal-framework-for-medical-scientific-research/your-research-is-it-subject-to-the-wmo-or-not).

**Fig. 1:**
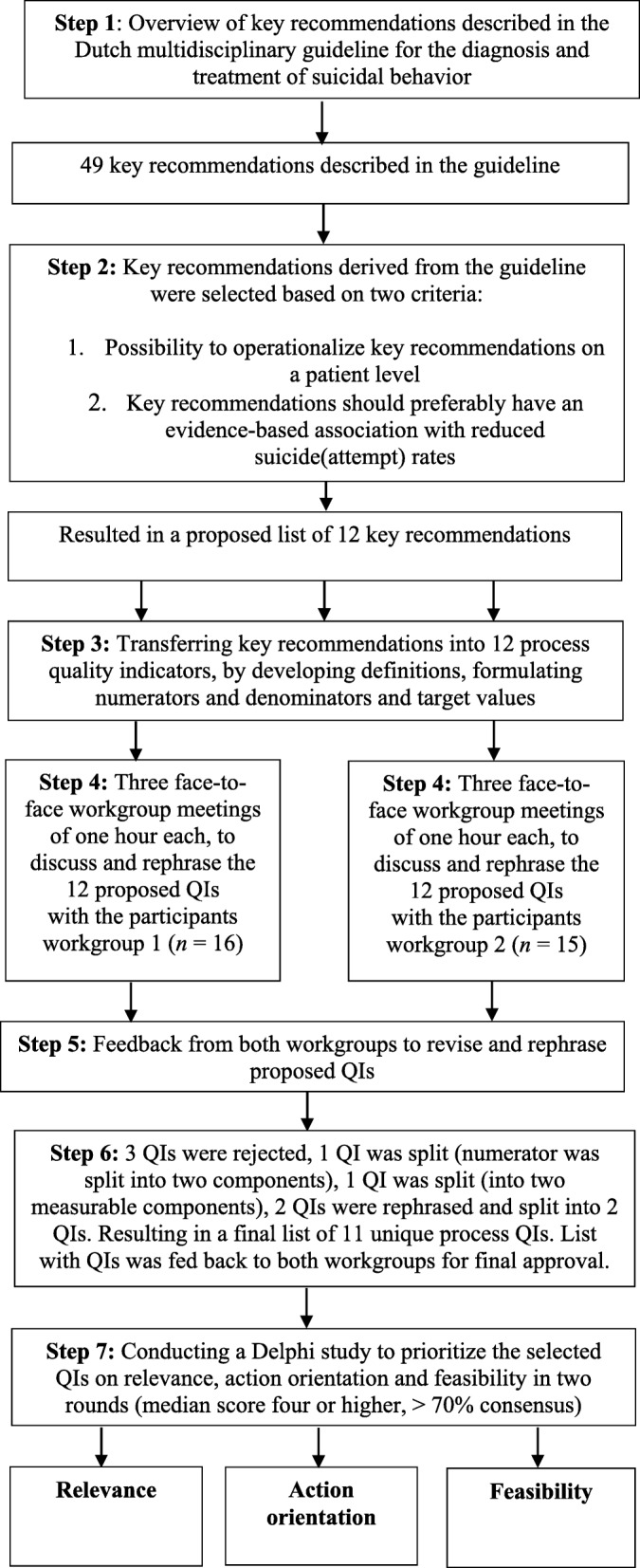
Flowchart of Delphi study with procedure for QI development

For this study, the SQUIRE 2.0 checklist (Standards for Quality Improvement Reporting Excellence) was used as a reporting guideline [27]. The procedure for the development of the QIs is shown in Fig. 1 and described in the procedure section below.

### Procedure

The procedure was conducted in two phases:

- *Phase one: Development and selection of guideline recommendations and QIs*

From the 49 key recommendations described in the suicide prevention guideline, an inventory of potential recommendations was selected (Fig. 1). These key recommendations were selected based on the possibility to operationalize them on a patient level. Furthermore, it was preferred that there was an evidence-based association between the key recommendations and reduced suicide (attempt) rates. This procedure resulted in a selection of 12 key recommendations from the guideline.

The selected key recommendations were modified into 12 process QIs (on a patient level) by describing them into detail, including definitions, numerators, and denominators and target values (see Additional files). Next, two small multidisciplinary workgroups of healthcare professionals (workgroup one; *n* = 16; workgroup two; *n* = 15) discussed the list of 12 QIs. During three sessions of one hour each, participants in both workgroups were instructed to discuss the 12 proposed QIs, based on their knowledge and expertise in the field. Furthermore, the participants appraised each QI individually with an overall score, reflecting whether the study should reject the QIs or rephrase them.

As illustrated in Fig. 1, participants rejected two QIs the Collaborative Assessment & Management of Suicidality (CAMS) and the Chronological Assessment of Suicide Events (CASE approach) due to low feasibility. Also, the participants in the workgroups argued that both indicators are not yet sufficiently implemented into routine care. The QI social connectedness was rejected by the participants due to a lack of scientific evidence. Also, the participants stated that social connectedness showed much overlap with other QIs and categorized this as a structural QI instead of an indicator that should be measured on a patient level. The numerator of the QI involvement of family or significant others was split into two components: A) there is contact with family or significant others and B) contact person is registered in patients’ electronic medical record (Additional files). Next, one QI was split into two QIs because the participants stated that the QI EHealth actually contains two important measurable components: 1) active usage and 2) availability of EHealth (Additional files). Finally, the participants also split the QI continuity of care: members of both workgroups agreed to divide this indicator into two QIs continuity of care and follow-up after discharge. Between each workgroup session, QIs were further clarified by the first author (KS) in terms of their clarity and definition. This resulted in a final set of 11 defined and operationalized QIs (Additional files). After final approval from the participants in both workgroups, the Delphi study was performed (phase two).

- *Phase two: Rating of guideline recommendations and QIs (Delphi study)*

The Delphi study consisted of two rounds:

### Round one: Rating QIs on relevance and action orientation

To achieve convergence of opinion, we developed an anonymous electronic survey with the 11 selected key recommendations and their QIs (Additional file 1). All MHIs participating in the SUPRANET network were involved in this study. Participants working in the field of suicide prevention were recruited from each MHI. Participants were approached in the first round via e-mail to complete the survey (Additional file 1) by following a web link. Written consent of participants was given by filling in the survey.

A total of 90 participants recruited from each MHI (23 suicide experts, 23 members of patients’ advisory boards or experts with experiences in suicidal behavior and 44 health care professionals), filled in the survey. They rated the QIs independently on two aspects: relevance (it affects the number of suicides in the institution) and action orientation (institutions or professionals themselves can influence it). All participants rated the QIs on both aspects using a 5-point Likert-scale (1 = strongly disagree, 2 = disagree, 3 = neither disagree or agree, 4 = agree and 5 = strongly agree).

### Round two: Rating QIs on feasibility

In the second round, members with specific expertise on data such as data analysts working in MHIs (*n* = 6), independently rated the QIs on feasibility (is it feasible to monitor and extract from existing systems) using an online survey (Additional file 2). The QIs were scored on a 5-point Likert scale (1 = not feasible at all, 2 = not feasible, 3 = neither not feasible or feasible, 4 = feasible and 5 = completely feasible). After both Delphi rounds, outcomes were analyzed, resulting in a specific set of relevant, action-oriented and feasible QIs.

### Data analyses

Analysis of both rounds of the Delphi study yielded consensus results by computing medians, and percentage of consensus for each item to determine which QIs achieved positive consensus. Data of respondents were anonymized prior to analysis. Agreement percentages were calculated for each item by assessing if at least 70% of the participant response rates were within the range of the median scores of four and five. The level of consensus achieved concerning the 11 QIs included in the first round was assessed using the following cut-off score [24, 28]:

1. Consensus = median score of four or higher on both selection criteria (relevance and action orientation) with > 70% consensus.
2. No consensus = median score of four or higher with < 70% consensus for one of the selection criteria (relevance or action orientation).
3. Not suitable = median score of three or lower for one of the selection criteria (relevance or action orientation).

Median scores and percentage of consensus was analyzed between the three groups of participants: 1) suicide experts (*n* = 23), 2) members of patients’ advisory boards and experts with experiences in suicidal behavior (*n* = 44) and 3) health care professionals (*n* = 23). Analyses were performed with SPSS version 24.0.

## Results

### Participants

The Delphi survey was sent to 154 experts by e-mail, and a total of 90 experts filled in the online questionnaire (response percentage of 58%). Of the 90 participants, 23 people (25.6%) were suicide experts, 23 (25.6%) were members of patients’ advisory boards or experts with experiences in suicidal behavior, and 44 (48.9%) were healthcare professionals. Almost all participants were from the Netherlands (*n* = 88), two participants lived in Belgium. From the participants that filled in the Delphi survey (*N* = 90), the mean age of the participants was 48.6 (*SD* = 11.39) years, of which 58.9% (*n* = 53) was female. The mean years of experience of healthcare professionals in Dutch mental healthcare was 18.10 years (*SD* = 9.73).

### Round one: Rating  QIs on relevance and action orientation

In the first round of the Delphi study, participants reached a consensus regarding the relevance for eight of the 11 QIs, and five of the 11 QIs were rated as action-oriented (Table 1). The participants scored the following five QIs that belonged to the guideline as both relevant and action-oriented: 1) screening suicidal thoughts and behavior, 2) safety plan, 3) early follow-up on discharge, 4) continuity of care, and 5) involving family or significant others. As Table 1 shows, between-group differences for relevance and action orientation were found for several prioritized QIs (screening, continuity of care, early follow-up on discharge, and involving family or significant others). Participants reached consensus on relevance, and action orientation for QIs highlighted in bold.

**Table 1:** Percentage of consensus for both relevance and action orientation (round one)

	Quality indicators	Members of patients’ advisory boards or experts with experiences in suicidal behavior (*n* = 23)				Healthcare professionals (*n* = 44)				Suicide experts (*n *= 23)				Total (*N* = 90)					Results
		Relevance		Action orientation		Relevance		Action orientation		Relevance		Action orientation		Relevance		Action orientation		Target value	(round one)
		consensus (%)	median score	consensus (%)	median score	consensus (%)	median score	consensus (%)	median score	consensus (%)	median score	consensus (%)	median score	consensus (%)	median score	consensus (%)	median score		
1	Availability of EHealth	65	4,0	38,1	3,0	50	3,5	48,7	3,0	59,1	4,0	47,6	3,0	56,1	4,0	45,7	3,0	100%	Rejected
2	Actively usage of EHealth	52,6	4,0	52,6	4,0	52,5	4,0	48,7	3,0	68,2	4,0	61,9	4,0	56,8	4,0	53,2	4,0	100%	Rejected
3	Screening suicidal thoughts and behavior^a^	90,9	5,0	81,8	4,0	68,3	5,0	69,2	4,0	63,6	4,0	63,6	4,0	72,9	5,0	71,1	4,0	100%	**Accepted**
4	Safety plan^a^	86,9	5,0	78,3	5,0	80,9	5,0	75,6	4,0	86,9	5,0	82,6	4,0	84,1	5,0	78,2	4,0	100%	**Accepted**
5	Waiting list duration	80	5,0	84,2	5,0	70,7	4,0	52,5	4,0	71,4	4,0	33,3	3,0	73,2	4,0	55	4,0	100%	Rejected
6	Early follow-up on discharge^a^	78,3	5,0	69,6	5,0	82,9	5,0	72,5	4,0	91,3	4,0	69,6	4,0	83,9	5,0	70,9	4,0	100%	**Accepted**
7	Continuity of care^a^	91,3	5,0	78,3	4,0	88,1	5,0	80	4,0	75	4,0	65,2	4,0	85,2	5,0	75,6	4,0	100%	**Accepted**
8A	There is contact with family or significant others^a^	78,3	5,0	73,9	5,0	90,5	4,0	75,6	4,0	78,3	5,0	69,6	4,0	84,1	5,0	73,6	4,0	100%	**Accepted**
8B	Contact person is registered in patients’ electronical medical record^a^	82,6	5,0	73,9	5,0	80,9	4,0	80,5	4,0	72,7	4,0	68,2	4,0	79,3	4,0	75,6	4,0	100%	**Accepted**
9	Structural diagnosis	72,7	4,0	63,6	4,0	75,6	4,0	64,1	4,0	75	4,0	55	4,0	74,7	4,0	61,7	4,0	100%	Rejected
10	Evidence-based medication	63,6	4,0	52,4	4,0	60,5	4,0	58,3	4,0	68,2	4,0	45	3,0	63,4	4,0	53,2	4,0	100%	Rejected
11	Evidence-based psychotherapy	69,6	5,0	68,2	4,0	74,4	4,0	62,2	4,0	78,3	4,0	63,6	4,0	74,1	4,0	64,2	4,0	100%	Rejected

(%) responses

^a^Quality indicator that achieved consensus during first round (consensus was achieved if median score was at least four or higher with > 70% consensus among participants)

No consensus was reached on relevance and action orientation for three QIs: 1) availability of EHealth, 2) active usage of EHealth, and 3) evidence-based medication. Further, 1) structural diagnosis, 2) waiting list duration, and 3) evidence-based psychotherapy achieved consensus for relevance but were not rated as action-oriented by the participants.

### Round two: Rating QIs on feasibility

During the second round of the Delphi study, data analysts (*n* = 6) rated the QIs on feasibility (Table 2). The percentage of consensus and median scores for the rated QIs on feasibility are shown in Table 2. When looking at the consensus cut-off score, participants rated only one of the 11 QIs as feasible. Early follow-up after discharge was prioritized as the only relevant, action-oriented, and feasible QI. For the QI waiting list duration, participants reached borderland consensus. As for the other nine QIs, no consensus on feasibility was reached. Both rounds of the Delphi study resulted in a final list of relevant, actionable and feasible QIs derived from the guideline. These results are presented in Table 3.

**Table 2:** Percentage of consensus and median scores for feasibility (round two). Percentage in bold achieved consensus

Prioritization quality indicators on feasibility				
	Quality indicators	Total (*n* = 6)		Results (round two)
		Consensus (%)	Median score	
1	Availability of EHealth	33,3	2,0	Rejected
2	Actively usage of EHealth	40,0	3,0	Rejected
3	Screening suicidal thoughts and behavior	16,7	1,0	Rejected
4	Safety plan	33,3	2,0	Rejected
5	Waiting list duration	66,7	4,0	Rejected
6	Early follow-up on discharge^a^	100,0	4,0	**Accepted**
7	Continuity of care	20,0	2,5	Rejected
8A	There was contact with family or significant others	50,0	3,5	Rejected
8B	Contact person is registered in patients’ electronical medical record	33,3	4,5	Rejected
9	Structural diagnosis	20,0	2,5	Rejected
10	Evidence-based medication	0,0	2,5	Rejected
11	Evidence-based psychotherapy	0,0	3,0	Rejected

(%) responses

^a^Quality indicator that achieved consensus during second round (consensus was achieved if median score was at least four or higher with > 70% consensus among participants)

**Table 3:** Final list of relevant, actionable and feasible quality indicators derived from the guideline

Selection prioritized quality indicators			
	Relevance	Action oriented	Feasibility
1	Continuity of care	Safety plan	Early follow-up on discharge
2^a^	Safety plan	Contact person is registered in patients’ electronical medical record (indicator 8B)	.
	There is contact with family or significant others (indicator 8A)	Continuity of care	
3	Early follow-up on discharge	There is contact with family or significant others (indicator 8A)	.
4	Contact person is registered in patients’ electronical medical record (indicator 8B)	Screening on suicidal thoughts and behavior	.
5	Screening on suicidal thoughts and behavior	Early follow-up on discharge	.

Top three was calculated using cut-off median score of four or higher, with highest % consensus

^a^Similar percentage of consensus was reached for both quality indicators

## Discussion

This study aimed to generate a set of relevant, action-oriented, and feasible QIs derived from the guideline by using a Delphi method. Only one QI was rated by six Dutch data analysts as feasible to monitor and register in MHIs in the Netherlands and reached consensus on all three criteria (relevancy, action orientation, feasibility). This QI was early follow-up on discharge. According to the literature, the risk of suicide is three times higher in the first week after discharge from a psychiatric facility [29] and remains significantly higher during the next few months [30, 31] or even years [32]. While the study of Zalsman et al. [13] reported that there is insufficient evidence for the efficacy of supportive contacts after discharge from the emergency department (ED), a large UK-study of Kapur et al. [2] found an association between rapid follow-up contact after inpatient discharge and lower suicide rates in mental healthcare services. Also, Luxton et al. [33] argued that brief supportive contacts by phone, text, postcards, or letters could be effective in reducing suicide (attempts) during high-risk periods (i.e., hospitalization or ED visits). Even though more high-quality RCTs are still needed to examine its impact further, numerous studies [14, 22, 34, 35], quality standards and suicide prevention guidelines [3, 36] repeatedly recommend improving early follow-up contact after discharge. However, whether a QI is rated as feasible will differ between institutions and countries.

Following the total group of experts, consensus on relevance (it affects the number of suicides in the institution), and action orientation (the institutions or professionals themselves can influence it) was reached on the following four QIs:

I. *Screening of suicidal thoughts and behavior*

Based on our results, the QI screening of suicidal thoughts and behavior was rated as a high priority. While some clinicians hold a belief that asking about suicide could trigger a patient into a suicidal act [37], a recent review found no iatrogenic effects [38]. An extensive systematic review of Zalsman et al. [13] reported inconclusive results on the costs and effects of routine screening and its association with reduced suicide (attempt) rates. Results from other studies also found that screening instruments are not very accurate in identifying the risk of suicide attempts or suicide death in individuals [39, 40]. A study by Coffey [41], however, showed that routine screening could be a useful and feasible method if screening results are regularly fed back to care teams and if it is included in the registration systems of MHIs. Although more empirical research on the effects of routine screening is needed, it is perceived by the literature as a very helpful strategy [22, 34, 35, 42]. To effectively treat individuals whose risk of suicide is high, it is very important to identify those individuals. Although evidence shows that predicting suicide with certainty is rather complex, it is not necessary to perfectly predict suicide in order to intervene effectively. For example, the National Action Alliance for Suicide Prevention [42] argues that by identifying individuals with an elevated risk, it allows mental healthcare professionals to detect those high-risk individuals who could then be treated with adequate and effective interventions.

II. *Safety plan*

Safety plan was rated as an essential QI for mental healthcare. The Dutch multidisciplinary suicide prevention guideline and care quality standards recommend regular and collaborative safety planning for all patients at risk of suicide [3, 43]. Also, safety plans are embedded in cognitive behavioral therapy (CBT) for suicide prevention [44]. Literature shows that there is some empirical evidence on the effectiveness of safety plans in reducing suicide (attempt) rates. For example, an RCT study of Bryan et al. [20] examined the impact of crisis response safety planning on suicide (attempts) among US soldiers at high-risk of suicide. The authors found a significant reduction of suicide ideation, fewer hospitalization days, and a 76% reduction in suicide attempts. These results are in line with a cohort comparison study of Stanley et al. [21], where a significant association between a Safety Planning Intervention (SPI) and reduced suicidal behavior was found in veteran hospitals. It should be noted, however, that this study design is limited since both intervention and control emergency department sites were matched instead of being randomized. Furthermore, both studies were performed in a veteran population. The results from both studies on the impact of safety plans are promising, but more RCT studies on its impact, also in other study populations, are highly needed.

III. *Continuity of care*

Consensus was achieved for continuity of care as a relevant andaction-oriented QI. In daily practice, it often happens that more than one clinician treats a patient at risk of suicide in a short period of time [3]. Transfer between clinicians, settings, or organizations could affect the continuity of care and is a well-known risk factor for patients at risk of suicide [45]. It is, therefore, important that clinicians collaborate and inform each other to ensure these patients receive the care they need. A few years ago, a report was published containing information about how continuity of care should be organized in Dutch mental healthcare [46]. This report aimed to stimulate and improve continuity of care by formulating recommendations (i.e., following suicide prevention training) to healthcare professionals (including general practitioners, medical specialists, emergency doctors). The literature highly recommends minimizing discontinuities in care for patients at risk of suicide during critical phases (i.e., transfers, post-discharge) [3, 34, 35]. However, more empirical research on its impact is needed. Fortunately, the scientific  evidence is building. For example, the study of Kapur et al. [2] found a significant association between the implementation of policies aiming at optimizing continuity of care for patients at risk of suicide in mental healthcare services and a reduction in the number of suicides . The importance of close communication between clinicians and across mental healthcare settings is also emphasized by other literature [22, 47]. Other studies should try to replicate the results of Kapur et al. [2] to further determine the impact of continuity of care on reduced suicide rates in mental healthcare.

IV. *Involving family or significant others*

Although the impact of involving family, carers or friends on suicide rates is rather understudied in the literature, this strategy on itself is highly recommended by the Dutch multidisciplinary suicide prevention guideline and various quality standards [3, 48]. Relatives can play an essential role in the prevention of suicide, but only if they are capable of supporting the mental healthcare services in the early detection and management of family members at risk [49]. They can provide valuable information to clinicians that could be helpful for the patients’ treatment. The importance of actively involving and informing family members is also highlighted in other studies [50, 51]. However, it is important to take into account that patients should always be allowed to say whether they would like their family or friends to be involved in their treatment or not [48]. A clinician should bear in mind the patients’ expressed wishes and views in relation to sharing information with their family, carers, or friends. On the contrary, relatives of a patient should be supported and provided with information as well. Based on a report of the department of Health and Social Care in England, relatives and carers have repeatedly raised their concerns about mental healthcare services who seem reluctant to take information from families or give them information about a patients’ suicide risk (https://assets.publishing.service.gov.uk/government/uploads/system/uploads/attachment_data/file/271792/Consensus_statement_on_information_sharing.pdf). It is of great importance to address these concerns, given the fact that the collaboration between a therapist and patients’ relatives is one of the components which can lead to better quality and safety improvement in specialist mental healthcare [34].

### Not prioritized guideline recommendations

Some guideline recommendations and their QIs were not endorsed as priorities, including evidence-based medication and psychotherapy for patients in specialist mental healthcare. This is surprising given the fact that both medication (lithium, clozapine, ketamine) and psychotherapy have shown promising results in reducing suicidality among patients in mental healthcare [13, 15–17].

It is possible that because medication and psychotherapy are already embedded in routine care, both recommendations were considered to be of less priority. Furthermore, participants in this study reported that medication and psychotherapy could not be influenced directly or changed by the MHIs or the healthcare professionals themselves, leading to a lower score on action orientation. Between-group differences were found for several guideline recommendations. For example, the expert group with members of patients’ advisory boards or experts with experiences in suicidal behavior rated screening as highly relevant and action-oriented, whereas the other two groups reached borderland consensus for this guideline recommendation. Also, the suicide expert group did not reach an agreement on action orientation for several recommendations and their QIs: 1) early follow-up on discharge, 2) continuity of care, and 3) involving family or significant others. This is in contrast with the outcomes of the other two participating expert groups, who did succeed in achieving consensus on action orientation for the same recommendations.

Guideline implementation is intended to result in a higher quality of mental healthcare, eventually leading to lower suicide rates. To achieve this goal, scientific evidence suggests that an optimal approach is required for implementing a combination of multiple relevant and action-oriented guideline recommendations at the same time. For example, a large UK-study examined the association between the implementation of key mental health service recommendations and suicide rates [23]. They found that services that had implemented seven to nine recommendations had a significantly lower suicide rate than those implementing fewer recommendations [23]. These findings suggest that the effort of implementing recommendations in mental healthcare services can affect suicide rates.

### Strengths and limitations

A key strength of this study is that experts from MHIs were continuously involved in both the development and selection of guideline recommendations and their QIs. Another strength was the breadth of expertise of the participants who participated in the Delphi study. Also, this survey sample (*N* = 90) was notably larger compared to other Delphi studies in the field of mental health [28]. The size of a Delphi panel is generally under 50, although more have been employed [25]. As for both rounds, the survey response rate was good overall. Nevertheless, there are some limitations. First, the participants in this study mainly came from the Netherlands. The established set of QIs might not match MHIs in other countries, and this applies in particular to the feasibility of the QIs. Second, the survey did not offer an opportunity for the 90 experts to suggest QIs that were not derived from the suicide prevention guideline.

## Conclusions

This study stands to contribute to the scientific literature on prioritizing relevant and action-oriented suicide prevention guideline recommendations to improve the quality of care in specialist mental healthcare. The results of this study are an important step towards the use of a new set of relevant and actionable QIs that are clearly defined, operationalized, and contain target values, making them appropriate for benchmarking and monitoring guideline implementation in specialist mental healthcare. If needed, other countries can tailor the selected QIs to one’s own (specialist) mental healthcare setting.

The prioritized QIs will be used to monitor the degree of guideline implementation within the specialist MHIs [6]. The five guideline recommendations selected as relevant and action-oriented might be of most importance for suicide prevention when implemented altogether. The SUPRANET study [6] will evaluate the implementation process of the prioritized recommendations in MHIs in the Netherlands. When proven to be effective, MHIs should include the prioritized recommendations into their policy for suicide prevention.

## Supplementary information

**Additional file 1:** Questionnaire round 1 of the Delphi study, to achieve convergence of opinion. Quality indicators were rated by the participants on two selection criteria: relevance and action orientation

**Additional file 2:** Questionnaire round 2 of the Delphi study. Quality indicators were rated by the participants on feasibility

## Data Availability

The datasets used and/or analyzed during this study will be made available after approval from the corresponding author or the SUPRANET board on reasonable request.

## Abbreviations

CAMS: Collaborative Assessment & Management of Suicidality intervention
CASE: Chronological Assessment of Suicide Events
MHI: Mental healthcare institution
PHQ: Patient Health Questionnaire-9
QI: Quality indicator
SQUIRE: Standards for Quality Improvement Reporting Excellence
SUPRANET: Suicide PRevention Action NETwork
