# Supplementary material for: Prioritizing suicide prevention guideline recommendations in specialist mental healthcare: a Delphi study
Source: BMC Psychiatry. 2020 Feb 7;20:55. doi: 10.1186/s12888-020-2465-0 (PMC7007653; doi:10.1186/s12888-020-2465-0)
Supplement: Supplementary file 2 — Additional file 2: Questionnaire round 2 of the Delphi study. Quality indicators were rated by the participants on feasibility [file 12888_2020_2465_MOESM2_ESM.docx]

**Appendix II.**

**Round two of the Delphi study**

Dear Registration Group member,

This questionnaire is about quality indicators for measuring and improving suicide prevention in the mental health service.

The quality indicators are indicative of the care provided and how it is organized. They are the ‘knobs’ that we need to turn in the expectation of producing better outcomes – in our case, fewer suicide attempts and fewer suicides on the part of patients being treated in mental health facilities. A group of professionals, including yourself, have now drawn up a list of possible quality indicators. These outcomes were analysed.

What we would like you to do

We would like to invite you, in your capacity as a member of the Registration Group to be involved in the next step in this development process, scoring the selected indicators on feasibility. This will provide us with a limited, supported set of indicators.

How the questionnaire works

You are asked to rate each of the eleven indicators on a scale from 1 to 5 in terms of the extent to which the indicator is feasible in your opinion.

- Feasibility: How easy is this indicator to collect at patient level?

Using your input and that from the other members of the Registration Group, we shall prioritize the set of provisional indicators.

Thank you in advance for completing the questionnaire.

Name

Demographic details

Below we ask a few general questions about yourself.

Gender

 Male

 Female

How old are you?

How long have you been working in mental health care?

Questionnaire

Eleven indicators are set out below. For each indicator we give the definition formulated by the Quality of Care Group of SUPRANET, the scientific literature and the Dutch multidisciplinary guideline on suicidal behaviour.* You should rate the indicators based on feasibility.

For each criterion you should select the appropriate rating on a scale of 1 to 5.

** Multidisciplinary guideline on suicidal behaviour (van Hemert et al., 2012).*

Indicator 1: Availability for eHealth focusing on suicidality

**Definition**: The use of digital information and communication technologies, or online treatment with one or more treatment aspects, focusing specifically on suicidal ideation and freely accessible to all patients in the mental health service, with the aim of supporting the care provided and improving patients’ health as regards suicidal ideation.

Measurement method

**Numerator**: The total number of patients with suicidal ideation/behaviour at a facility who were being treated (in an outpatient and/or clinical setting) at any time during the data collection period and had access to an eHealth self-help programme focusing on suicidal ideation during that period.

**Denominator**: The total number of patients at a facility who were being treated (in an outpatient and/or clinical setting) during the same data collection period.

Your opinion:

The scale runs from 1 = not feasible at all to 5 = highly feasible

|  | **Do not know** | **1** | **2** | **3** | **4** | **5** |
| --- | --- | --- | --- | --- | --- | --- |
| Feasibility |  |  |  |  |  |  |

Indicator 2: Active use of eHealth focusing on suicidality

**Definition**: The use of digital information and communication technologies, or online treatment with one or more treatment aspects, focusing specifically on suicidal ideation and freely accessible to all patients in the mental health service, with the aim of supporting the care provided and improving patients’ health as regards suicidal ideation.

Measurement method

**Numerator**: The total number of patients with suicidal ideation/behaviour at a facility who were being treated (in an outpatient and/or clinical setting) at any time during the data collection period and had actively made use of an eHealth self-help programme focusing on suicidal ideation during that period.

**Denominator**: The total number of patients at a facility who were being treated (in an outpatient and/or clinical setting) during that data collection period.

Your opinion:

The scale runs from 1 = not feasible at all to 5 = highly feasible

|  | **Do not know** | **1** | **2** | **3** | **4** | **5** |
| --- | --- | --- | --- | --- | --- | --- |
| Feasibility |  |  |  |  |  |  |

Indicator 3: Screening for suicidal thoughts and behaviour

**Definition**: All patients, during any type of treatment contact, are asked* about the presence and severity of suicidal thoughts and behaviours.

*(* Examples of possible questions about suicidal ideation during a screen: Have you had suicidal thoughts during the past month? If so, what are your reasons for dying and what are your reasons for living? How strong is your intent to commit suicide? Have you made any preparations?)*

Measurement method

**Numerator**: The number of individual treatment contacts with all patients during the data collection period at which they were demonstrably asked about the presence and severity of suicidal thoughts and behaviours.

**Denominator**: The total number of treatment contacts with all patients during the same data collection period.

Your opinion:

The scale runs from 1 = not feasible at all to 5 = highly feasible

|  | **Do not know** | **1** | **2** | **3** | **4** | **5** |
| --- | --- | --- | --- | --- | --- | --- |
| Feasibility |  |  |  |  |  |  |

Indicator 4: Safety plan

**Definition**: A safety plan is an early warning plan, care plan or treatment plan, drawn up jointly by a patient and his or her care provider, that focuses specifically on safety from suicidal ideation.

Measurement method

**Numerator**: The total number of patients for whom at any time during treatment (in an outpatient and/or clinical setting) a safety plan focusing on suicidal ideation was recorded in their medical records. A safety plan must meet the following criteria:

- The safety plan drawn up jointly by the patient and the therapist is no more than one year old.
- The safety plan focuses on suicidal ideation.
- A third party is involved in the safety plan.
- The safety plan for a patient who has suicidal ideation or has attempted suicide was updated at the most recent treatment contact.

**Denominator**: The total number of patients at a facility who were being treated (in an outpatient and/or clinical setting) during the same data collection period.

Your opinion:

The scale runs from 1 = not feasible at all to 5 = highly feasible

|  | **Do not know** | **1** | **2** | **3** | **4** | **5** |
| --- | --- | --- | --- | --- | --- | --- |
| Feasibility |  |  |  |  |  |  |

Indicator 5: Waiting time

Definition based on the Supranet Care minimum data set*

For each patient who had his or her first treatment contact (T2) during the data collection period, the length of time between registration (T0) and the first treatment contact (T2).**

- *T0 is the time of registration.*
- *T1 is the time of intake.*
- *T2 is the time of the first treatment contact between the patient and the therapist.*

Measurement method

**Numerator**: The total number of patients who had their first treatment contact during the data collection period, with a maximum of two weeks between registration and the first treatment contact.***

**Denominator**: The total number of patients who had their first treatment contact during the same data collection period.

** The minimum data set is the core records of Supranet Care. This data set contains variables that need to be collected in order to obtain a baseline for feedback. The current data set includes such things as sociodemographic details (gender, age, marital status), information on the care provided (treatment setting, treatment duration, principal diagnosis), organizational parameters (total length of stay, total number of days at the facility with/without overnight stay, number of psychiatric beds), and the number of suicides and suicide attempts.*

*** Treatment of patients suspected of mental incapacity must always start on the day they are registered.*

**** Calendar day of both registration and first treatment contact known.*

Your opinion:

The scale runs from 1 = not feasible at all to 5 = highly feasible

|  | **Do not know** | **1** | **2** | **3** | **4** | **5** |
| --- | --- | --- | --- | --- | --- | --- |
| Feasibility |  |  |  |  |  |  |

Indicator 6: Early follow-up on discharge

**Definition**: The patient should have been in contact with the care provider again within two weeks of discharge from the facility. Contact can be treatment or counselling by or feedback to the same care provider. This must be personal, face-to-face contact, and may take place outside the facility if appropriate.

Excluded are patients who have been referred back or referred to their GP (or practice support worker), front-line mental health care or a facility other than their (integrated) facility.

Measurement method

**Numerator**: The number of patients who during the data collection period, following discharge from the facility, had a face-to-face follow-up contact with that facility.

**Denominator**: The total number of patients discharged from the facility during the same data collection period.

Your opinion:

The scale runs from 1 = not feasible at all to 5 = highly feasible

|  | **Do not know** | **1** | **2** | **3** | **4** | **5** |
| --- | --- | --- | --- | --- | --- | --- |
| Feasibility |  |  |  |  |  |  |

Indicator 7: Continuity of care

**Definition**: Transfer of the patient from one therapist to another, preceded by a verbal consultation between the two therapists, with a transition or change in the patient’s current care allocation.

Measurement method

**Numerator**: The total number of patients who had a transfer preceded by a verbal consultation from one therapist to another during the data collection period.

**Denominator**: The total number of patients being treated at the facility who were transferred during the same data collection period.

Your opinion:

The scale runs from 1 = not feasible at all to 5 = highly feasible

|  | **Do not know** | **1** | **2** | **3** | **4** | **5** |
| --- | --- | --- | --- | --- | --- | --- |
| Feasibility |  |  |  |  |  |  |

Indicator 8a: Involvement of family or significant others

**Definition**: In the case of all patients there was contact with one or more loved ones at the start of, during and at the end of treatment, or (in the event of objections on the part of the patient or loved ones) achieving this was a goal of treatment. A loved one is anyone who is part of the patient’s support system.

Measurement method

**Numerator 1**: The number of patients being treated (in an outpatient and/or clinical setting) during the data collection period in whose case there was contact with one or more loved ones during and at the end of treatment.

**Denominator**: The total number of patients who were being treated (in an outpatient and/or clinical setting) during the same data collection period.

Your opinion:

The scale runs from 1 = not feasible at all to 5 = highly feasible

|  | **Do not know** | **1** | **2** | **3** | **4** | **5** |
| --- | --- | --- | --- | --- | --- | --- |
| Feasibility |  |  |  |  |  |  |

Indicator 8b: Involvement of family or significant others

**Definition**: In the case of all patients there was contact with one or more loved ones at the start of, during and at the end of treatment, or (in the event of objections on the part of the patient or loved ones) achieving this was a goal of treatment. A loved one is anyone who is part of the patient’s support system.

Measurement method

**Numerator 2**: The number of patients being treated (in an outpatient and/or clinical setting) during the data collection period in whose case a current contact person was entered in the patient’s electronic medical record.

**Denominator**: The total number of patients who were being treated (in an outpatient and/or clinical setting) during the same data collection period.

Your opinion:

The scale runs from 1 = not feasible at all to 5 = highly feasible

|  | **Do not know** | **1** | **2** | **3** | **4** | **5** |
| --- | --- | --- | --- | --- | --- | --- |
| Feasibility |  |  |  |  |  |  |

Indicator 9: Structural diagnosis

**Definition**: A structural diagnosis is a diagnosis that is as descriptive and explanatory as possible of a patient’s suicidal state during the past month (thoughts, intentions, plans, motives). It should include at least the nature, intensity and consequences of suicidal behaviour, perpetuating, protective and risk factors, and the patient’s mental capacity as regards suicide.

Measurement method

**Numerator**: The total number of patients during the data collection period who had suicidal ideation and at any time during treatment (in an outpatient and/or clinical setting) had a structural diagnosis that meets the following criteria:

- The structural diagnosis was recorded in the electronic medical record (EMR) during treatment.
- The current structural diagnosis is no more than one year old, and in the case of repeated attempts it has been updated during treatment.
- During the course of the illness the structural diagnosis has been reviewed regularly to see whether any factors in it have changed over time.

**Denominator**: The total number of suicidal patients* at a facility who were being treated (in an outpatient and/or clinical setting) at any time during the same data collection period.

* i.e. patients with suicidal thoughts and/or behaviour.

Your opinion:

The scale runs from 1 = not feasible at all to 5 = highly feasible

|  | **Do not know** | **1** | **2** | **3** | **4** | **5** |
| --- | --- | --- | --- | --- | --- | --- |
| Feasibility |  |  |  |  |  |  |

Indicator 10: Evidence-based medication

**Definition**: Drug treatment for patients with a depressive disorder who display suicidal ideation as a syndrome. The drug treatment is designed to reduce suicidal ideation in the patient and aims to prevent suicidal thoughts and suicide attempts. The drug treatment is rational and applied in line with the guidelines or based on other considerations.

Measurement method

**Numerator**: The number of patients with a depressive disorder (in an outpatient and/or clinical setting) who had suicidal ideation as a syndrome of the psychiatric disorder and received evidence-based drug treatment during the data collection period.

**Denominator**: The total number of patients with a depressive disorder (in an outpatient and/or clinical setting) who had suicidal ideation as a syndrome of the psychiatric disorder during the same data collection period.

Your opinion:

The scale runs from 1 = not feasible at all to 5 = highly feasible

|  | **Do not know** | **1** | **2** | **3** | **4** | **5** |
| --- | --- | --- | --- | --- | --- | --- |
| Feasibility |  |  |  |  |  |  |

Indicator 11: Evidence-based psychotherapy

**Definition**: Psychotherapy for a depressive disorder, in which the patient has suicidal ideation as a syndrome of the psychiatric disorder, designed to directly influence the severity of the suicidal behaviour and reduce it. The treatment includes promoting both safety and the working relationship, treating psychiatric dysregulation, determining the correct setting for treatment and promoting continuity of care. The psychotherapy is applied in line with the guidelines* or based on other considerations.

Measurement method

**Numerator**: The number of patients with a depressive disorder (in an outpatient and/or clinical setting) who had suicidal ideation as a syndrome of the psychiatric disorder and received evidence-based psychotherapy during the data collection period.

**Denominator**: The total number of patients with a depressive disorder (in an outpatient and/or clinical setting) who had suicidal ideation as a syndrome of the psychiatric disorder during the same data collection period.

Your opinion:

The scale runs from 1 = not feasible at all to 5 = highly feasible

|  | **Do not know** | **1** | **2** | **3** | **4** | **5** |
| --- | --- | --- | --- | --- | --- | --- |
| Feasibility |  |  |  |  |  |  |

Of the eleven indicators that you have rated, please list the top three that you consider to be most feasible.

Number 1 (most important)

Number 2

Number 3
